# Mid‐ to long‐term outcomes of capsular management in hip arthroscopy for FAIS: A multilevel meta‐analysis

**DOI:** 10.1002/ksa.70259

**Published:** 2025-12-31

**Authors:** Nikolai Ramadanov, Maximilian Voss, Robert Hable, Robert Prill, Roland Becker, Ingo J. Banke

**Affiliations:** ^1^ Center of Orthopaedics and Traumatology, Brandenburg Medical School University Hospital Brandenburg an der Havel Brandenburg an der Havel Germany; ^2^ Faculty of Health Science Brandenburg, Brandenburg Medical School Theodor Fontane Brandenburg an der Havel Germany; ^3^ Faculty of Applied Computer Science, Deggendorf Institute of Technology Deggendorf Germany; ^4^ Clinic of Orthopaedics and Sports Orthopaedics, School of Medicine and Health TUM University Hospital Technical University of Munich Munich Germany; ^5^ AGA‐Society for Arthroscopy and Joint‐Surgery, Hip Committee, c/o Walder Wyss Ltd. Zürich Switzerland

**Keywords:** capsular management, capsule preservation, capsule repair, femoroacetabular impingement syndrome, hip arthroscopy, long‐term

## Abstract

**Purpose:**

To compare mid‐ to long‐term outcomes of the three major capsular management strategies—capsule preserved (CP), capsule repaired (CR) and capsule unrepaired (CU)—following hip arthroscopy (HAS) for femoroacetabular impingement syndrome (FAIS).

**Methods:**

A systematic search was conducted in PubMed, Embase, CENTRAL, and Epistemonikos for studies published up to 31 May 2025. Studies were eligible if they reported ≥2‐year outcomes after HAS with clearly described capsular techniques. Outcomes included the modified Harris Hip Score (mHHS), Hip Outcome Score—Activities of Daily Living (HOS‐ADL) and Sports Subscale (HOS‐SSS) and Visual Analogue Scale (VAS) for pain. Multilevel meta‐analyses were performed using random‐effects models with Hartung–Knapp adjustment. Failure‐related outcomes (complications, reoperations, total hip arthroplasty [THA] conversions) were inconsistently reported across studies and could only be summarised descriptively.

**Results:**

Seven studies with a total of 948 patients were included. Pooled mean mHHS was 81.14 (95% confidence interval [CI]: 77.72–84.56), with CP: 84.90, CR: 80.66 and CU: 80.25 (*p* = 0.67). HOS‐ADL pooled mean was 87.94 (95% CI: 84.79–91.09), with CP: 89.30, CR: 87.36, CU: 88.31 (*p* = 0.90). HOS‐SSS pooled mean was 78.59 (95% CI: 72.69–84.48), with CP: 75.70, CR: 78.89, CU: 80.22 (*p* = 0.84). VAS pooled mean was 2.42 (95% CI: 2.08–2.77), with CP: 2.46, CR: 2.27, CU: 2.76 (*p* = 0.51). No statistically significant subgroup differences were detected. CR showed the highest cumulative numbers of reoperations and THA conversions, whereas CP and CU demonstrated lower but heterogeneous event counts.

**Conclusion:**

CP, CR and CU demonstrated comparable mid‐ to long‐term patient‐reported outcome measure (PROM) outcomes following HAS for FAIS. Failure‐related events varied descriptively across techniques, but inconsistent reporting prevented comparative survivorship assessment.

**Level of Evidence:**

Level II, systematic review and multilevel meta‐analysis of predominantly Level III studies, with additional contributions from Level I and II studies.

**Abbreviations**
ADL: Activities of Daily Living Subscale
BDDH: borderline developmental dysplasia of the hip
BMI: body mass index
CI: confidence interval
CP: capsule preserved
CR: capsule repaired
CU: capsule unrepaired
DAA: direct anterior approach
FAI: femoroacetabular impingement
HAGOS: Copenhagen Hip and Groin Outcome Score
HAS: hip arthroscopy
HOOS: Hip Disability and Osteoarthritis Outcome Score
HOS: Hip Outcome Score
iHOT: International Hip Outcome Tool
*KSSTA*
: *Knee Surgery, Sports Traumatology, Arthroscopy*
MCID: minimal clinically important difference
mHHS: modified Harris Hip Score
NAHS: Non‐Arthritic Hip Score
NRS: Numeric Rating Scale
PRISMA: Preferred Reporting Items for Systematic Reviews and Meta‐Analyses
PROM: patient‐reported outcome measure
PROSPERO: International Prospective Register of Systematic Reviews
RCT: randomized controlled trial
RoB: Risk of Bias
ROBINS: Risk Of Bias In Non‐randomised Studies of Interventions
SSS: Sports Subscale
THA: total hip arthroplasty
VAS: Visual Analogue Scale

## INTRODUCTION

Hip arthroscopy (HAS) requires deliberate management of the hip capsule as a key step in the effective treatment of labral, ligamentum teres and femoroacetabular pathologies. Optimal capsular handling is considered essential for preserving joint stability, optimizing outcomes, and minimizing complications [45]. Surgical approaches vary and include: (i) capsule preserved (CP)—no formal capsulotomy, access via periportal techniques alone; (ii) capsule repaired (CR)—formal capsulotomy followed by repair with sutures or plication and (iii) capsule unrepaired (CU)—formal capsulotomy left open [40]. Evidence suggests periportal techniques may maintain stability without repair, while full repair reduces microinstability risk [45].

Several meta‐analyses have assessed capsular management in HAS, particularly for femoroacetabular impingement syndrome (FAIS). While early work by Ortiz‐Declet et al. [75] suggested benefits of capsular closure or plication, no pooled statistics were reported. Subsequent analyses by Liu et al. [58] and Lin et al. [57] found no consistent differences between closure and non‐closure groups, with some heterogeneity and limited evidence of instability. More recent studies by Cohen et al. [16], Looney et al. [60] and Dasari et al. [22] reported improved functional outcomes and reduced revision rates with complete capsular closure, though limitations such as short follow‐up and variability in techniques remained. Phillips et al. [84] compared closure and non‐closure but excluded CP approaches. A recent and comprehensive multilevel meta‐analysis by Ramadanov et al. [87], published in *Knee Surgery, Sports Traumatology, Arthroscopy* (*KSSTA*), which included 47 primary studies encompassing 7366 hips, provides the most robust evidence to date on this topic. The analysis demonstrated that both CP and capsule‐repair (CR) strategies lead to superior short‐ and mid‐term functional outcomes compared to unrepaired capsulotomy (CU), with no consistent differences between CP and CR. These findings support CP and CR as preferable strategies over CU, while still allowing for individualized surgical decisions based on patient anatomy and intraoperative factors. Notably, long‐term outcomes remain to be established. To date, no study has clarified the long‐term consequences of CP, CR and CU. Prior evidence is almost entirely short‐term, leaving unanswered whether early differences persist, diminish, or translate into complications or arthroplasty risk. This gap underscores the clinical need for a focused mid‐ to long‐term meta‐analysis.

Long‐term outcomes are essential in orthopaedics because many surgical interventions aim to provide durable symptom relief, preserve joint function, and prevent future procedures such as total joint arthroplasty. Short‐ and mid‐term improvements may not capture delayed complications, functional decline, or the true impact on joint longevity. Therefore, a separate meta‐analysis focusing on mid‐ to long‐term outcomes is warranted to guide evidence‐based decision‐making and assess the sustained benefits of different capsular management strategies.

The aim of this study was to perform a systematic review and multilevel meta‐analysis to compare mid‐ to long‐term clinical outcomes across the three principal capsular management strategies in HAS—CP, CR and CU.

## METHODS

### Reporting standards and protocol registration

This multilevel meta‐analysis was conducted using the same study protocol, prospectively registered in the International Prospective Register of Systematic Reviews (PROSPERO) on 10 May 2025 (CRD420251050197), as our previously published multilevel meta‐analysis [87]. Because short‐ and long‐term postoperative outcomes represent biologically distinct recovery phases and are reported by largely non‐overlapping studies, the present analysis was conducted separately from our previously published short‐term multilevel meta‐analysis [87], published in *KSSTA*. This systematic review and multilevel meta‐analysis followed the updated Preferred Reporting Items for Systematic Reviews and Meta‐Analyses (PRISMA) guidelines [77]. A completed PRISMA checklist is provided in Table S1.

### Information sources and search strategy

A literature search was performed across PubMed, Embase, the Cochrane Central Register of Controlled Trials (CENTRAL), and Epistemonikos to identify studies published up to 31 May 2025. Customized search strategies were developed for each database using Boolean operators to capture studies related to HAS and capsular management. The core search terms included combinations of: ((capsule) OR (capsulotomy) OR (capsular repair) OR (capsular closure) OR (capsular management)) AND ((hip arthroscopy) OR (arthroscopy) OR (HAS)). No filters were applied for language or date of publication.

### Eligibility criteria

Studies were eligible for inclusion if they were randomized controlled trials (RCTs), prospective or retrospective observational studies, or case series that specifically evaluated defined capsular management techniques. Case reports, narrative reviews, and editorials were excluded. Acceptable interventions included: (i) periportal techniques involving small capsular openings limited to a few mm, such as portal dilation, expansion, or puncture capsulotomy (typically sub‐centimetre in size, matching instrument width); (ii) interportal or T‐shaped capsulotomies with capsular opening typically ranging from 2.5 to 6 cm, followed by repair using approximately 3–8 side‐to‐side or shoelace sutures and (iii) similar capsulotomies performed without subsequent repair. Primary studies that reported outcomes for only one capsular management technique and did not include comparative capsular groups were nevertheless eligible because the multilevel meta‐analytic framework allows single‐arm contributions to individual technique levels (CP, CR, CU). This approach avoids selection bias and increases the precision of technique‐specific estimates without requiring within‐study comparisons. Studies were excluded if they: (i) did not report mid‐ to long‐term outcomes (defined as ≥2 years post‐surgery); (ii) included mixed treatment groups without stratified results; (iii) failed to adequately describe the capsular intervention; (iv) omitted key functional or pain‐related hip outcomes; (v) presented overlapping patient samples (in which case the most complete or recent dataset was selected) or (vi) focused on capsular plication in the context of borderline hip dysplasia for stability purposes.

### Study selection and data extraction

Two reviewers (N.R. and M.V.) independently conducted the study selection process in two stages: initial screening of titles and abstracts, followed by full‐text evaluation. Any disagreements were resolved in consultation with a third reviewer (I.J.B.). Inter‐rater agreement was quantified using the kappa statistic (*κ*). Data extraction was likewise performed independently by the same reviewers, with discrepancies resolved by consensus involving additional reviewers as needed. Key data items extracted included study identifiers (author, year, country), design characteristics, sample size, participant demographics, follow‐up duration, risk of bias and relevant clinical outcomes. Primary outcomes were patient‐reported outcome measures (PROMs) assessing hip function, including the mHHS (modified Harris Hip Score) [44], HOS‐ADL (Hip Outcome Score—Activities of Daily Living) [64], HOS‐SSS (Hip Outcome Score—Sports Subscale) and hip pain, assessed by the Visual Analogue Scale (VAS) [51]. Data extraction for secondary outcomes included overall complications, deep vein thrombosis (DVT) or pulmonary embolism (PE), nerve injury, infection, reoperation and conversion to total hip arthroplasty (THA). All events were extracted exactly as reported in the primary studies and grouped according to capsular management technique.

### Study quality and bias assessment

Two reviewers (N.R. and M.V.) independently evaluated study quality and risk of bias. For RCTs, the Cochrane Risk of Bias 2 (RoB 2) tool [93] was applied, while non‐randomized studies were assessed using the ROBINS‐I tool [92]. Any disagreements were resolved through discussion with a third reviewer (I.J.B.). Potential publication bias was explored visually through funnel plots [33].

### Multilevel meta‐analysis

To estimate treatment effects among CP, CR and CU strategies, a multilevel meta‐analysis [81] was conducted using a frequentist random‐effects model with inverse‐variance weighting and restricted maximum likelihood (REML) estimation. The Hartung–Knapp adjustment was applied to improve robustness [86]. Mean values with 95% confidence intervals (CIs) were calculated for continuous functional outcomes within each group. Subgroup analyses were performed to test for statistically significant differences between groups. Between‐study heterogeneity was assessed using the Higgins *I* ^2^ statistic, classified as low (<25%), moderate (25%–75%), or high (>75%). Results were visualized in forest plots, and statistical significance was set at *p* < 0.05. All analyses were conducted using the meta and metafor packages in R by an experienced statistician (R.H.).

### Descriptive analysis

For failure‐related outcomes (complications, reoperations and conversion to THA), quantitative data synthesis was not feasible due to inconsistent reporting and substantial missing data across studies. Therefore, these outcomes were summarised descriptively for each capsular management technique without statistical pooling. Aggregated counts for overall complications, reoperations and THA conversions were extracted directly from the primary studies and are presented descriptively in the Results section.

## RESULTS

### Systematic literature search

**Figure 1:**
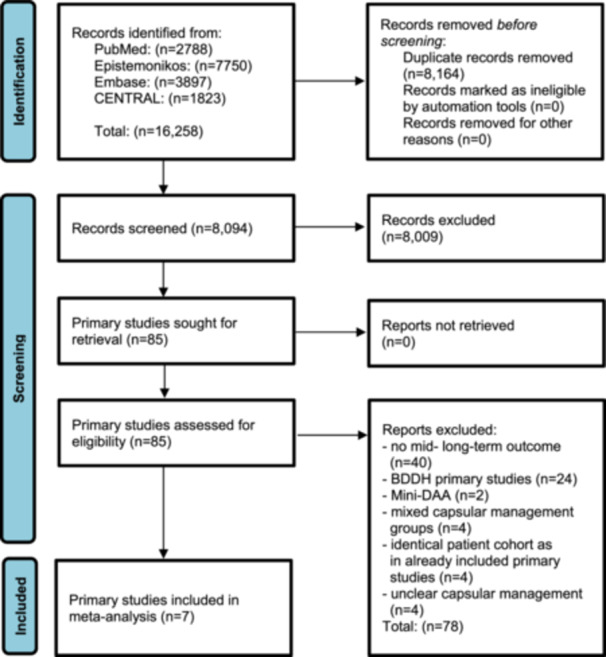
PRISMA chart flow diagram. BDDH, borderline developmental dysplasia of the hip; DAA, direct anterior approach; PRISMA, Preferred Reporting Items for Systematic Reviews and Meta‐Analyses.

A comprehensive literature search was conducted across four major databases—PubMed, Epistemonikos, Embase and CENTRAL—resulting in the identification of 16,258 unique records. After the removal of 8164 duplicates, a total of 8094 citations remained for initial screening based on titles and abstracts. Following this phase, 8009 records were excluded for not meeting the predefined eligibility criteria. Subsequently, 85 full‐text articles were reviewed in detail for potential inclusion [1, 2, 3, 4, 5, 6, 7, 8, 9, 10, 11, 12, 13, 14, 15, 17, 18, 19, 20, 21, 23, 24, 25, 26, 27, 28, 29, 30, 31, 32, 34, 35, 36, 37, 38, 39, 41, 42, 43, 46, 47, 48, 49, 50, 52, 53, 54, 55, 56, 59, 61, 62, 63, 65, 66, 67, 68, 69, 70, 71, 72, 73, 74, 76, 78, 79, 80, 82, 83, 85, 88, 89, 90, 91, 94, 95, 96, 97, 98, 99, 100, 101, 102, 103, 104], with no exclusions due to access limitations. Upon full‐text assessment, 78 studies were excluded for the following reasons: (i) lack of mid‐ to long‐term outcome data (*n* = 40) [3, 5, 7, 9, 12, 13, 18, 20, 24, 26, 32, 36, 42, 47, 48, 52, 54, 55, 56, 61, 63, 65, 67, 69, 70, 71, 73, 74, 76, 79, 82, 83, 88, 89, 91, 94, 95, 97, 98, 100]; (ii) exclusive focus on borderline developmental dysplasia of the hip (BDDH) populations (*n* = 24) [1, 2, 4, 6, 11, 14, 15, 21, 23, 34, 35, 38, 41, 43, 46, 59, 62, 66, 72, 85, 90, 99, 101, 103]; (iii) use of mini‐direct anterior approach (Mini‐DAA) techniques (*n* = 2) [17, 80]; (iv) mixed capsular management strategies without subgroup stratification (*n* = 4) [25, 37, 96, 102]; (v) overlapping patient cohorts with already included studies (*n* = 4) [19, 28, 29, 30] and (vi) unclear or unreported capsular procedures (*n* = 4) [10, 49, 68, 104]. Ultimately, seven primary studies [8, 27, 31, 39, 50, 53, 78] fulfilled all eligibility criteria and were included in the final meta‐analysis (see Figure 1).

### Study characteristics

A total of seven studies [8, 27, 31, 39, 50, 53, 78] were included in the final meta‐analysis, encompassing a range of study types and levels of evidence. Notably, all studies originated from the United States. Among these, one study was an RCT, classified as Level I evidence [50]. Five studies were retrospective cohort designs, contributing Level III evidence [8, 27, 31, 39, 53], while one prospective case series provided Level IV evidence [78]. All included investigations focused on patients undergoing HAS for FAIS [8, 27, 31, 39, 50, 53, 78]. Additionally, labral pathology in the context of FAIS was explicitly addressed in four of the studies [8, 27, 31, 39] (Table 1).

**Table 1:** Study characteristics.

Author	Origin	Journal	Study design	Level of evidence	Indication for HAS	Follow‐up duration
Bolia IK et al. 2019 [8]	USA	*The Journal of Arthroscopic and Related Surgey*	Retrospective cohort study	III	FAIS, labral lesion	≥60 months
Domb BG et al. 2018 [27]	USA	*Arthroscopy: The Journal of Arthroscopic and Related Surgery*	Retrospective cohort study	III	FAIS	≥60 months
Eberlin CT et al. 2023 [31]	USA	*The Orthopaedic Journal of Sports Medicine*	Retrospective cohort study	III	FAIS, labral lesion	≥24 months
Frank RM et al. 2014 [39]	USA	*The American Journal of Sports Medicine*	Retrospective cohort study	III	FAIS	~36 months
Krych AJ et al. 2013 [50]	USA	*Arthroscopy: The Journal of Arthroscopic and Related Surgery*	RCT	I	FAIS, labral lesion	≥24 months
Larson CM et al. 2024 [53]	USA	*Arthroscopy: The Journal of Arthroscopic and Related Surgery*	Retrospective cohort study	III	FAIS	≥24 months
Palmer DH et al. 2012 [78]	USA	*Arthroscopy: The Journal of Arthroscopic and Related Surgery*	Prospective case series	IV	FAIS, labral lesion	~42 months

Abbreviations: FAIS, femoroacetabular impingement syndrome; HAS, hip arthroscopy; RCT, randomized controlled trial.

### Patient characteristics

A total of 964 hips from 948 patients were included across the seven studies [8, 27, 31, 39, 50, 53, 78]. The mean patient age was 37 years, ranging from 33 to 40 years, with 44% males. The average body mass index (BMI) was 25 kg/m^2^, ranging from 24 to 26 kg/m^2^ (Table 2). The CP group included 348 patients (364 hips). The mean patient age was 39 years, ranging from 38–40 years, with 51% males. The average BMI was 26 kg/m^2^, ranging from 25 to 27 kg/m^2^. The CR group included 461 patients (461 hips). The mean patient age was 37 years, ranging from 33 to 39 years, with 43% males. The average BMI was 25 kg/m^2^, ranging from 24 to 26 kg/m^2^. The CU group included 139 patients (139 hips). The mean patient age was 36 years, ranging from 33 to 38 years, with 26% males. The average BMI was 24 kg/m^2^, ranging from 24 to 25 kg/m^2^.

**Table 2:** Patient characteristics.

Author	Capsular management	Capsular opening technique	Capsular closure technique	Operative treatment	Patients (hips), *N*	Age, years ± SD (range)	Male sex (%)	BMI, kg/m^2 ^± SD (range)
Bolia IK et al. 2019 [8]	CR	Linear interportal incision	Side‐to‐side absorbable sutures	Cam/Pincer resection, labral repair	84 (84)	38 ± 15	57	NR
	CU	Linear interportal incision	—		42 (42)	38 ± 15	57	NR
Domb BG et al. 2018 [27]	CR	Linear interportal/T‐shaped incision	Side‐to‐side non‐absorbable plication sutures	Cam/Pincer resection, labral repair, microfracture	65 (65)	38 ± 13	28	24.4 ± 3.8
	CU	Linear interportal/T‐shaped incision	—		65 (65)	37 ± 12	28	24.1 ± 3.3
Eberlin CT et al. 2023 [31]	CP	Puncture capsulotomy	—	Cam/Pincer resection, labral repair, microfracture	163 (163)	38 (36–40)	48	25.9 (25.2–26.5)
Frank RM et al. 2014 [39]	CR	Linear interportal and T‐shaped incision	Side‐to‐side non‐absorbable sutures	Cam/Pincer resection, labral repair, chondroplasty	32 (32)	33 ± 10	37	25.0 ± 3.2 (16.5–62.7)
	CU	Linear interportal and T‐shaped incision	Incomplete repair		32 (32)	33 ± 10	37	24.7 ± 3.8 (16.5–62.7)
Krych AJ et al. 2013 [50]	CR	T‐shaped incision	Side‐to‐side sutures	Cam/Pincer resection, labral repair	18 (18)	38 (20–59)	0	NR
	CR	T‐shaped incision	Side‐to‐side sutures		18 (18)	39 (19–55)	0	NR
Larson CM et al. 2024 [53]	CR	T‐shaped incision	Side‐to‐side non‐absorbable sutures	Cam/Pincer resection, labral repair	122 (122)	36 ± 12	0	24.9 ± 3.8
	CR	T‐shaped incision	Side‐to‐side non‐absorbable sutures		122 (122)	36 ± 11	100	25.7 ± 3.6
Palmer DH et al. 2012 [78]	CP	—	—	Cam/Pincer resection, labral repair, microfracture	185 (201)	40 (14–87)	54	NR
Total					948 (964)	37 (33–40)	44	25.0 (24.1–25.9)
CP					348 (364)	39 (38–40)	51	25.9 (25.2–26.5)
CR					461 (461)	37 (33–39)	43	25.0 (24.4–25.7)
CU					139 (139)	36 (33–38)	26	24.4 (24.1–24.8)

Abbreviations: BMI, body mass index; CP, capsule preserved; CR, capsule repaired; CU, capsule unrepaired; NR, not reported; SD, standard deviation.

### Quality assessment

**Figure 2:**
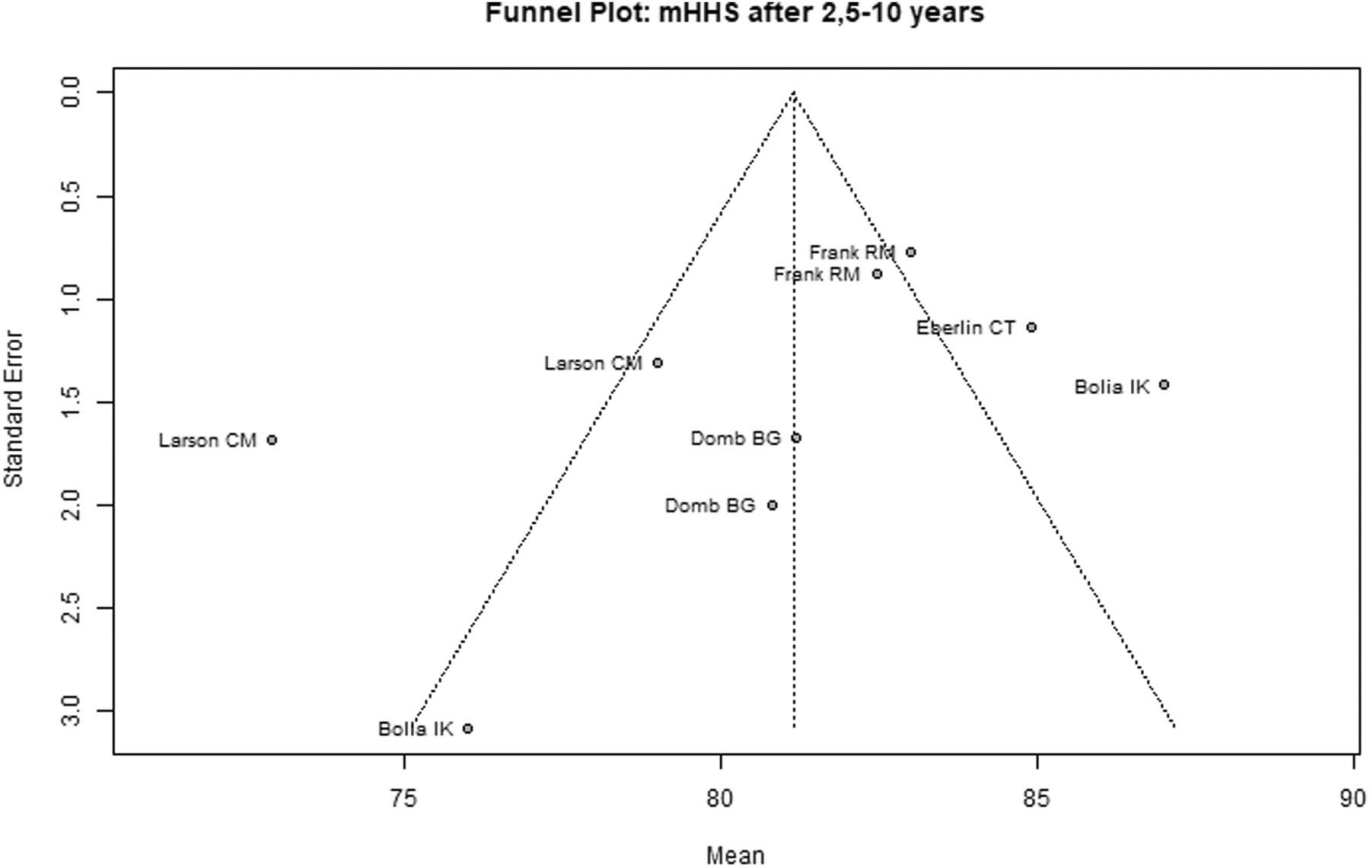
Funnel plot of the mHHS 2.5–10 years postoperatively. mHHS, modified Harris Hip Score.

**Figure 3:**
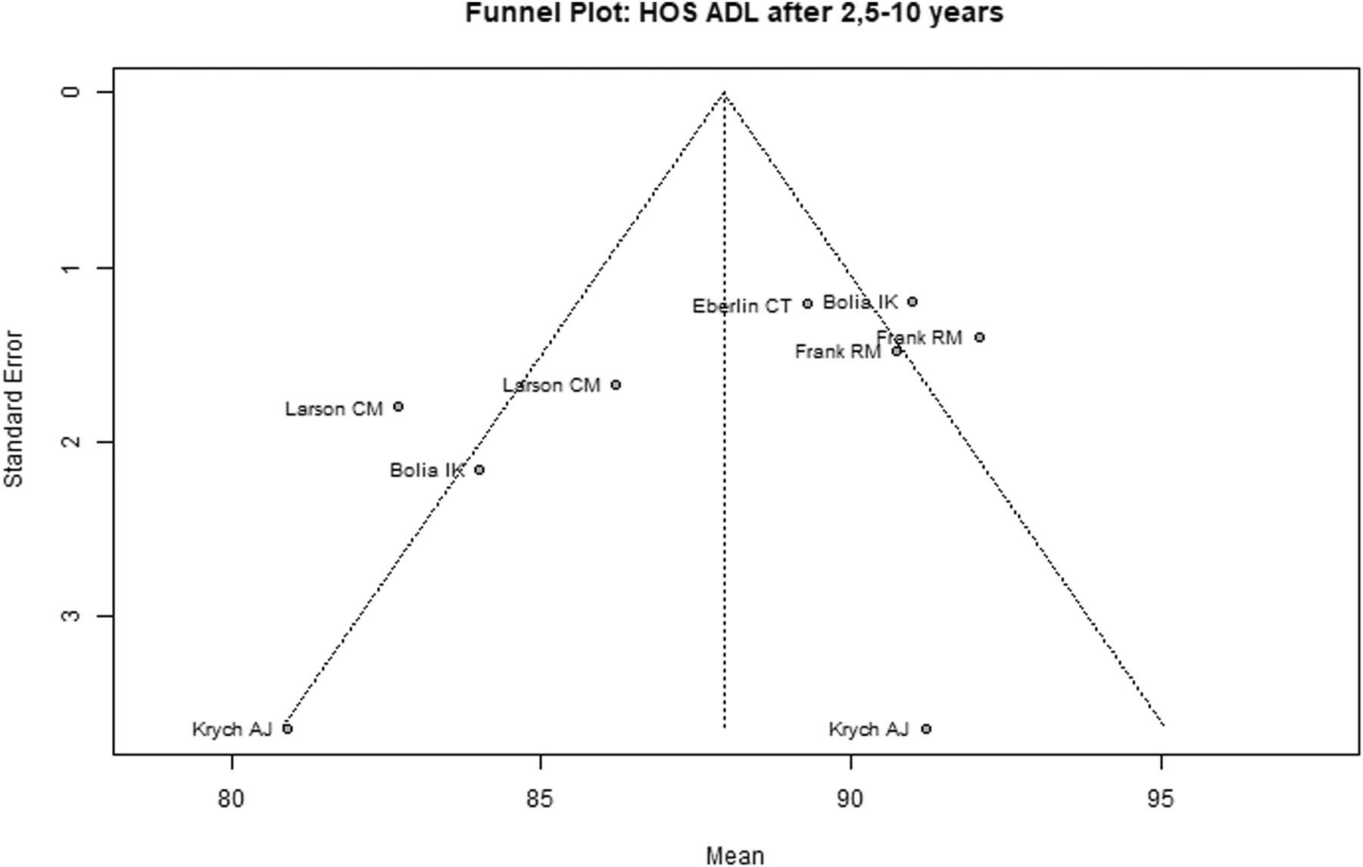
Funnel plot of the HOS ADL 2.5–10 years postoperatively. HOS‐ADL, Hip Outcome Score—Activities of Daily Living.

**Figure 4:**
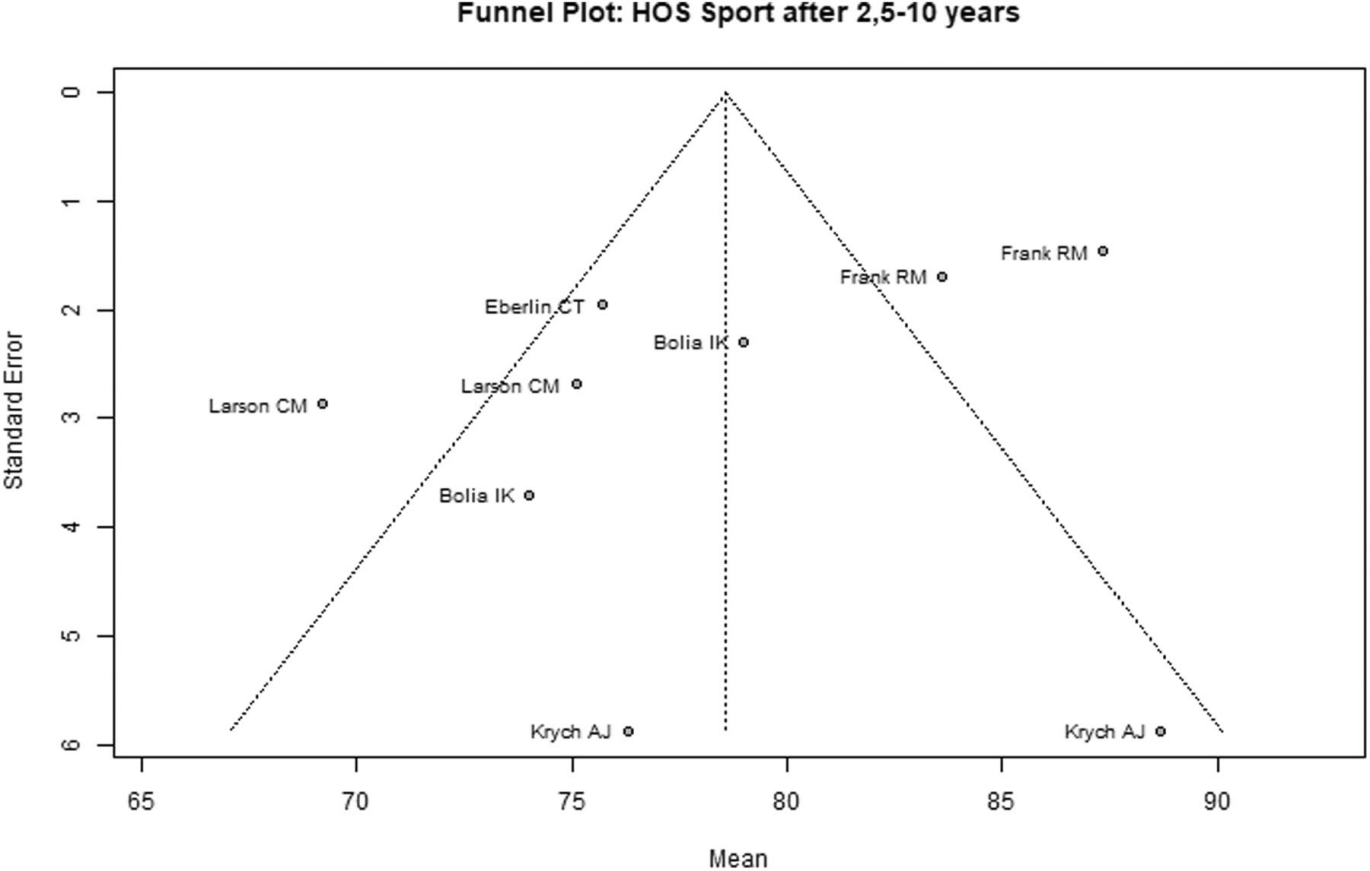
Funnel plot of the HOS SSS 2.5–10 years postoperatively. HOS‐SSS, Hip Outcome Score—Sports Subscale.

**Figure 5:**
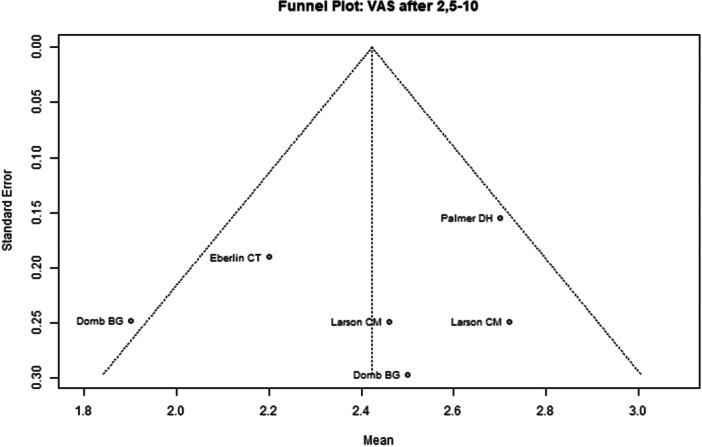
Funnel plot of the pain VAS 2.5–10 years postoperatively. VAS, Visual Analogue Score.

Among the seven included studies, two were judged to have a low overall risk of bias [50, 53], while the remaining five studies were assessed as having moderate risk [8, 27, 31, 39, 78] (Table 3). Visual inspection of the funnel plots (Figures 2, 3, 4, 5) revealed no clear evidence of publication bias. The plots for mHHS (Figure 2) and HOS ADL (Figure 3) appeared symmetric, suggesting a low likelihood of bias. Slight asymmetry was observed in the HOS SSS (Figure 4) and pain VAS (Figure 5) plots, indicating a potential for minor publication bias; however, this may also reflect heterogeneity or random variation.

**Table 3:** Risk of bias assessment using the ROBINS‐I tool for non‐RCTs and the RoB‐2 tool for RCTs.

Study	Bias due to confounding	Bias in selection of participants	Bias in classification of interventions	Bias due to deviations from intended interventions	Bias due to missing data	Bias in measurement of outcomes	Bias in selection of the reported result	Overall risk of bias
Bolia IK et al. 2019 [8]	Moderate	Low	Low	Low	Low	Low	Moderate	Moderate
Domb BG et al. 2018 [27]	Moderate	Low	Low	Low	Low	Low	Moderate	Moderate
Eberlin CT et al. 2023 [31]	Moderate	Low	Low	Low	Low	Low	Moderate	Moderate
Frank RM et al. 2014 [39]	Moderate	Low	Low	Low	Moderate	Low	Moderate	Moderate
Larson CM et al. 2024 [53]	Low	Low	Low	Low	Low	Low	Low	Low
Palmer DH et al. 2012 [78]	Moderate	Low	Low	Low	Moderate	Low	Moderate	Moderate

Study	Bias arising from the randomization process	Bias due to deviations from intended interventions	Bias due to missing outcome data	Bias in measurement of the outcome	Bias in selection of the reported result	Overall risk of bias
Krych AJ et al. 2013 [50]	Low	Low	Low	Low	Low	Low

Abbreviations: RCTs, randomized controlled trials; RoB, risk of bias; ROBINS‐I, Risk Of Bias In Non‐randomised Studies of Interventions.

### Mid‐ to long‐term postoperative outcome parameters

**Figure 6:**
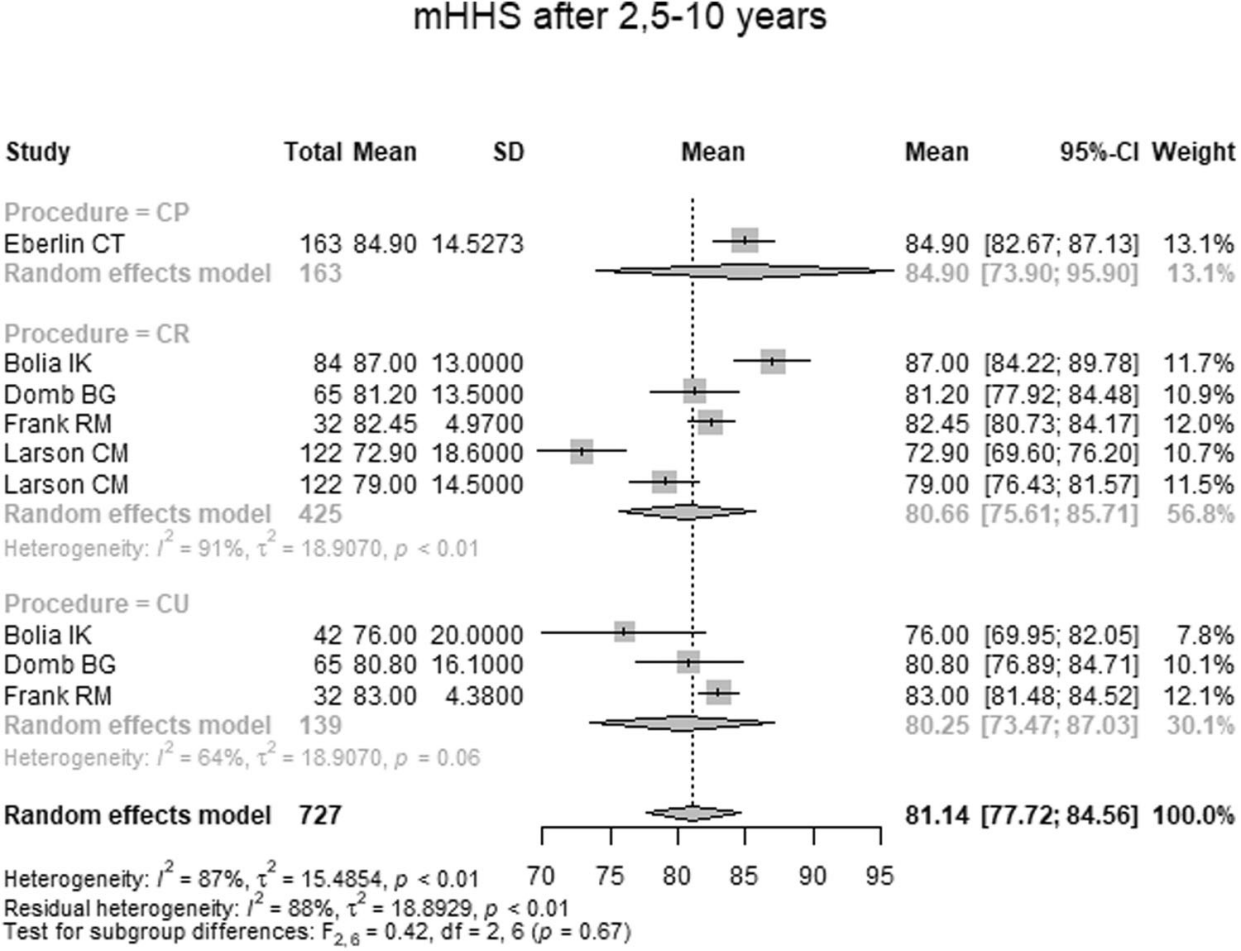
Forest plot of the mHHS 2.5–10 years postoperatively. CI, confidence interval; CP, capsule preserved; CR, capsule repaired; CU, capsule unrepaired; mHHS, modified Harris Hip Score; SD, standard deviation.

mHHS 2.5–10 years postoperatively was reported in five studies, including a total of 727 hips (Table 4 and Figure 6). The pooled mean was 81.14 (95% CI: 77.72–84.56). The mean in the CP group was 84.90 (95% CI: 73.90–95.90), in the CR group 80.66 (95% CI: 75.61–85.71) and in the CU group 80.25 (95% CI: 73.47–87.03). There was no statistically significant difference between groups (*p* = 0.67).

**Table 4:** Summary of the mid‐ to long‐term outcome parameters.

	Primary studies, *N*	Hips, *N*	Mean value	CIs	*τ* ^2^	*I* ^2^	Heterogenity *p*	Difference *p*
mHHS 2.5–10 years postoperatively								
Total	9	727	81.14	77.72; 84.56	15.49	0.87	<0.0001***	0.6724
CP	1	163	84.90	73.90; 95.90	18.91		—	
CR	5	425	80.66	75.61; 85.71	18.91	0.91	<0.0001***	
CU	3	139	80.25	73.47; 87.03	18.91	0.64	0.0626	
HOS ADL 2.5–10 years postoperatively								
Total	9	633	87.94	84.79; 91.09	10.90	0.77	<0.0001***	0.9036
CP	1	163	89.30	78.96; 99.64	16.39		—	
CR	6	396	87.36	82.74; 91.98	16.39	0.79	0.0002***	
CU	2	74	88.31	80.65; 95.97	16.39	0.90	0.0017**	
HOS SSS 2.5–10 years postoperatively								
Total	9	633	78.59	72.69; 84.48	29.68	0.86	<0.0001***	0.8413
CP	1	163	75.70	59.9; 91.5	37.89		—	
CR	6	396	78.89	70.99; 86.79	37.89	0.79	0.0002***	
CU	2	74	80.22	69.78; 90.66	37.89	0.91	0.0008***	
Pain VAS 2.5–10 years postoperatively								
Total	6	738	2.42	2.08; 2.77	0.05	0.52	0.0638	0.5054
CP	2	364	2.46	1.53; 3.38	0.14	0.76	0.0419*	
CR	3	309	2.27	1.31; 3.24	0.14	0.65	0.0581	
CU	1	65	2.76	1.43; 4.09	0.14		—	

Abbreviations: CIs, confidence intervals; CP, capsule preserved; CR, capsule repaired; CU, capsule unrepaired; mHHS, modified Harris Hip Score; HOS‐ADL, Hip Outcome Score—Activities of Daily Living; HOS‐SSS, Hip Outcome Score—Sports Subscale; VAS, Visual Analogue Score.

* Statistically significant.

** Very statistically significant.

*** Highly statistically significant.

**Figure 7:**
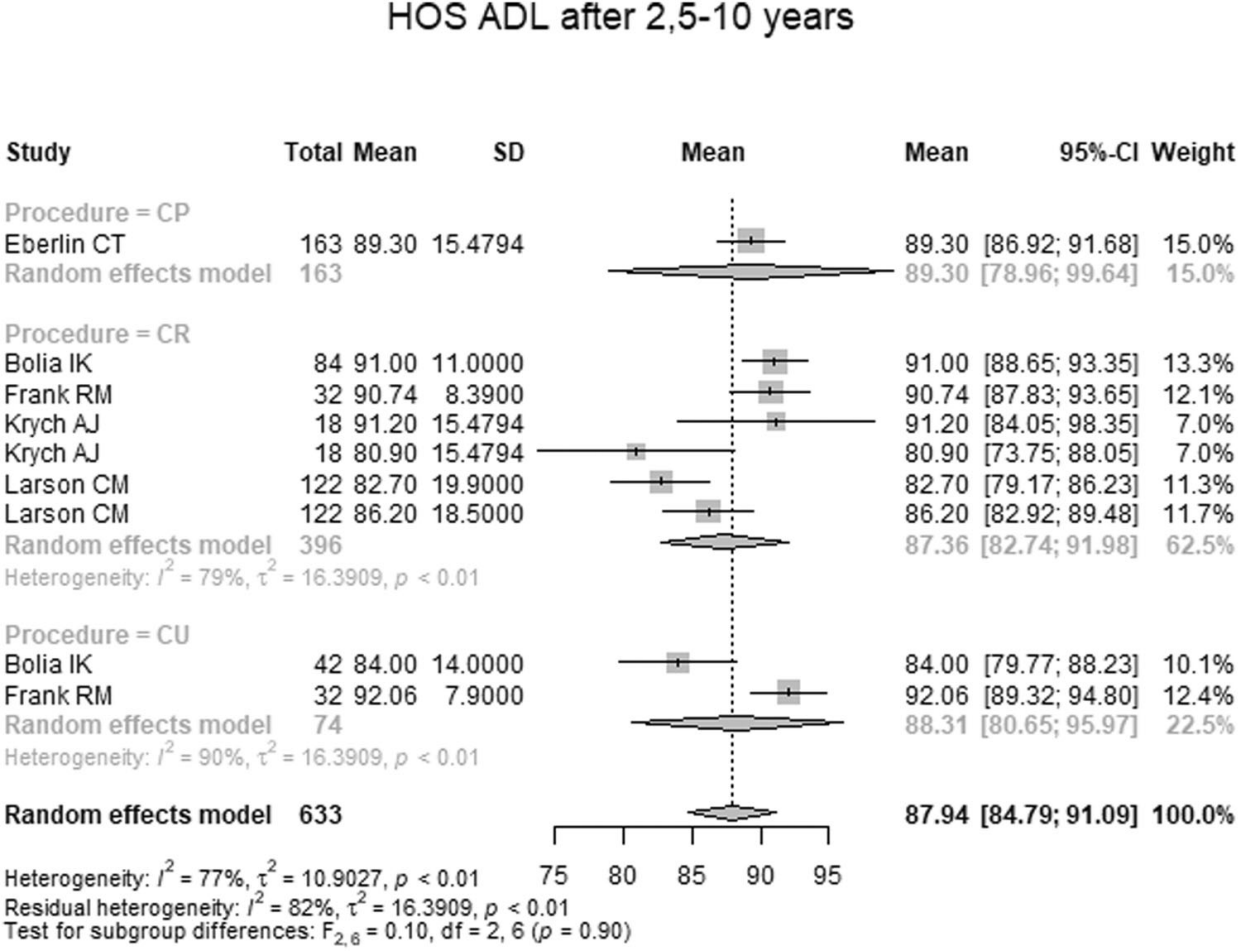
Forest plot of the HOS ADL 2.5–10 years postoperatively. CI, confidence interval; CP, capsule preserved; CR, capsule repaired; CU, capsule unrepaired; HOS‐ADL, Hip Outcome Score—Activities of Daily Living; SD, standard deviation.

HOS ADL 2.5–10 years postoperatively was reported in five studies with a total of 633 hips (Table 4 and Figure 7). The pooled mean was 87.94 (95% CI: 84.79–91.09). The mean in the CP group was 89.30 (95% CI: 78.96–99.64), in the CR group 87.36 (95% CI: 82.74–91.98), and in the CU group 88.31 (95% CI: 80.65–95.97). The difference between groups was not significant (*p* = 0.90).

**Figure 8:**
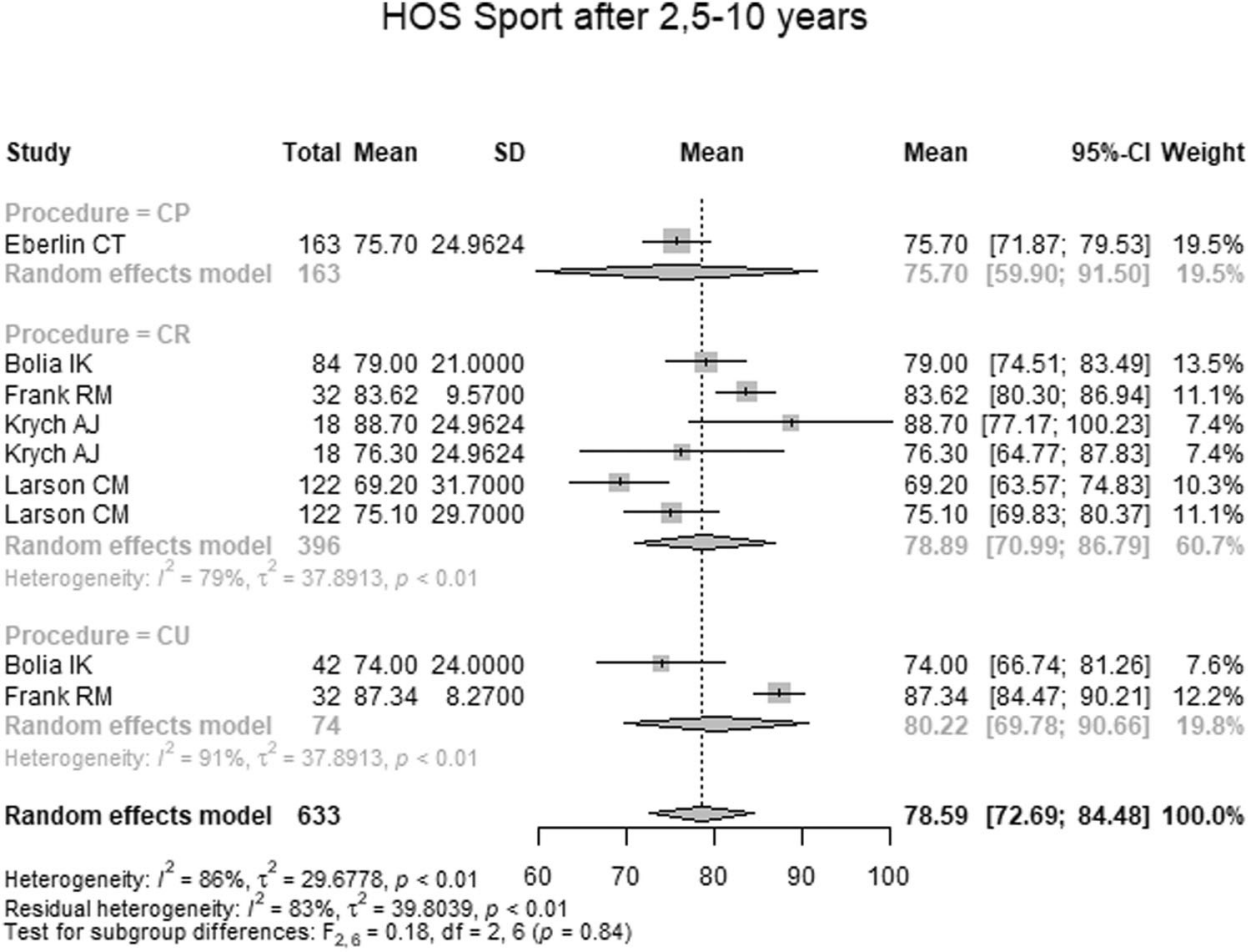
Forest plot of the HOS SSS 2.5–10 years postoperatively. CI, confidence interval; CP, capsule preserved; CR, capsule repaired; CU, capsule unrepaired; HOS‐SSS, Hip Outcome Score—Sports Subscale; SD, standard deviation.

HOS Sport 2.5–10 years postoperatively was reported in five studies with 633 hips (Table 4 and Figure 8). The pooled mean was 78.59 (95% CI: 72.69–84.48). The mean in the CP group was 75.70 (95% CI: 59.90–91.50), in the CR group 78.89 (95% CI: 70.99–86.79) and in the CU group 80.22 (95% CI: 69.78–90.66). No significant subgroup difference was detected (*p* = 0.84).

**Figure 9:**
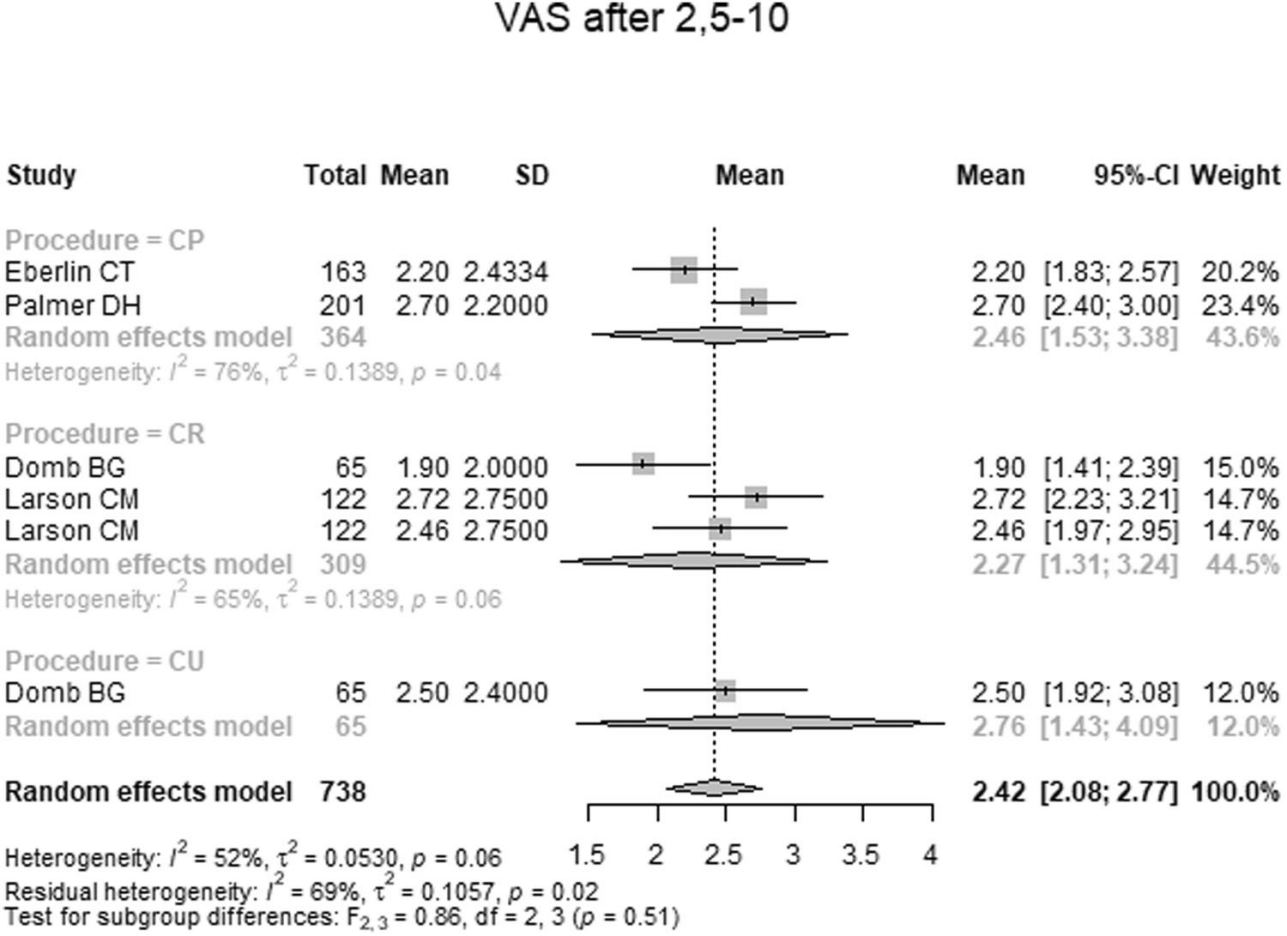
Forest plot of the pain VAS 2.5–10 years postoperatively. CI, confidence interval; CP, capsule preserved; CR, capsule repaired; CU, capsule unrepaired; SD, standard deviation; VAS, Visual Analogue Score.

Pain VAS 2.5–10 years postoperatively was reported in four studies comprising 738 hips (Table 4 and Figure 9). The pooled mean was 2.42 (95% CI: 2.08–2.77). The mean in the CP group was 2.46 (95% CI: 1.53–3.38), in the CR group 2.27 (95% CI: 1.34–3.21) and in the CU group 2.76 (95% CI: 1.43–4.09). No significant differences were found between groups (*p* = 0.51).

### Failure‐related outcomes

Failure‐related outcomes (complications, reoperations and conversion to THA) were descriptively summarised according to capsular management technique.

Overall, 23 complications were documented in two primary studies (19 in Eberlin et al. [31] and 4 in Palmer et al. [78]). These included three DVT/PE events, three nerve injuries and two infections. No reoperations were reported in either study [31, 78]. A total of 15 conversions to THA occurred, consisting of 2 cases in Eberlin et al. [31] and 13 cases in Palmer et al. [78], the latter representing the largest proportion of late failures within this group.

Complication and failure data for CR were available from Bolia et al. [8], Domb et al. [27], Frank [39] and Larson et al. [53]. A total of three overall complications were reported (Bolia et al. [8]; Domb et al. [27] and Frank et al. [39]), including two nerve injuries and one infection, with no documented DVT/PE events. Reoperations were more frequent in this group, with a cumulative 53 revision procedures: 10 in Bolia et al. [8], 4 in Domb et al. [27] and 39 in Larson et al. [53]. Similarly, conversion to THA was highest in the CR group, with 33 cases in total, including 12 in Bolia et al. [8], 0 in Domb et al. [27] and 21 in Larson et al. [53].

Failure‐related outcomes for CU were reported in two studies (Bolia et al. [8]; Domb et al. [27]). Combined, these studies documented four overall complications, including one DVT/PE event, two nerve injuries and no infections. A total of 14 reoperations were recorded (4 in Bolia et al. [8] and 10 in Domb et al. [27]). Conversion to THA occurred in 13 cases, consisting of 6 in Bolia et al. [8] and 7 in Domb et al. [27].

Across all included studies, CP demonstrated no reoperations but a notable number of THA conversions. CR showed the highest cumulative reoperation count (*n* = 53) and the highest number of THA conversions (*n* = 33). CU displayed intermediate rates, with 14 reoperations and 13 THA conversions.

## DISCUSSION

### Summary of main findings

This multilevel meta‐analysis of seven studies encompassing 826 patients provides the first focused evaluation of mid‐ to long‐term outcomes for the three different capsular management strategies currently used in HAS for FAIS. Across all assessed functional measures—mHHS, HOS ADL, HOS SSS and pain VAS—no statistically significant differences were found between the three main approaches: CP, CR and CU. While numerically higher scores were observed for CP and CR across several outcomes, these differences did not reach statistical significance, suggesting comparable long‐term efficacy among techniques when properly selected and executed.

This analysis was intentionally restricted to mid‐ and long‐term outcomes. The evidence base differs substantially from our prior short‐term multilevel meta‐analysis [87], with limited overlap in primary studies and distinct postoperative time windows. Combining both time periods within a single model would have introduced substantial temporal heterogeneity and obscured true long‐term effects.

### Discussion of these findings

The present findings contrast with prior short‐ and mid‐term analyses, including our own earlier multilevel meta‐analysis, which demonstrated significantly better early outcomes for CP and CR compared to CU [87]. In the current analysis, these advantages appear to diminish over time, as no statistically significant group differences were observed at 2.5–10 years postoperatively. One plausible explanation is the effect of long‐term biological adaptation: as capsular tissues heal and undergo remodelling, and periarticular structures regain neuromuscular control, early deficits in joint stability or proprioception may be progressively compensated. Additionally, postoperative rehabilitation and functional compensation may allow patients with CU to regain satisfactory performance levels over time. This theory is further supported by the uniformly low pain VAS scores and high functional scores observed across all groups, indicating that most patients ultimately experience favourable outcomes when the underlying pathology of FAIS is effectively treated. Nevertheless, the trend toward slightly lower scores in the CU group across most measures cannot be ignored and may still carry clinical importance in specific subpopulations, such as patients with ligamentous laxity or borderline hip morphology, who are more susceptible to capsular insufficiency and microinstability.

The descriptive analysis of failure‐related outcomes revealed notable differences between capsular techniques, although interpretation remains limited by inconsistent reporting and heterogeneous follow‐up durations across studies. CR demonstrated the highest cumulative numbers of reoperations and THA conversions, whereas CP showed no reoperations but several late THA conversions driven by individual cohorts. These findings suggest potential differences in long‐term mechanical stability between techniques, but the available evidence is insufficient for firm comparative conclusions. Future studies should report complications and reoperations more consistently to allow formal quantitative analysis.

### Comparison with related meta‐analyses

As in all HAS meta‐analyses, the surgical techniques in the included studies were also heterogeneous, and this inherent variability should be considered when interpreting our findings. Prior meta‐analyses by Ortiz‐Declet et al. [75], Liu et al. [58] and Lin et al. [57] provided early but often conflicting insights, hindered by limited sample sizes, heterogeneous surgical techniques and predominantly short‐term follow‐up data. More recently, large‐scale syntheses by Looney et al. [60] and Dasari et al. [22] demonstrated superior short‐term outcomes and reduced revision rates with CR. In line with these, our own earlier multilevel meta‐analysis [87], published in *KSSTA*, which focused on short‐ and mid‐term outcomes, showed significantly better functional improvement with CP and CR compared to CU. However, all of these prior efforts were constrained by relatively limited follow‐up durations (mostly ≤2 years). The present study complements and extends this body of evidence by isolating mid‐ to long‐term outcomes, thus providing a more stable and reliable view of the sustained effects of capsular strategy. Notably, the observed numerical similarity between CP and CR in the long term supports the emerging hypothesis that peripheral preservation techniques—defined as limited capsular access methods such as periportal approaches that preserve the majority of the capsular envelope and avoid large interportal or T‐capsulotomies—may offer comparable durability to formal repair, particularly when applied in appropriately selected patients.

### Anatomical considerations and clinical relevance

Capsular techniques interact with key anatomic stabilizers of the hip—particularly the iliofemoral ligament and the anterolateral capsular fibres—which differ in their contribution to rotational control and translational restraint. These structural differences may partly explain why CP and CR show numerically better long‐term stability profiles than CU in selected patients. However, because all included studies used heterogeneous surgical techniques, labral treatments and rehabilitation protocols, the generalizability of the pooled effects across broader patient populations (e.g., dysplastic or hypermobile hips) remains limited. Accordingly, our findings apply primarily to FAIS patients undergoing contemporary arthroscopic techniques in high‐volume North American centres.

### Practical implications and recommendations

Given the absence of significant long‐term outcome differences, the capsular strategy may be tailored to individual patient anatomy, pathology, and surgeon preference. For patients at high risk of instability (e.g., ligamentous laxity, shallow acetabulum), CR or CP should still be preferred. However, in low‐risk patients, especially when using minimally invasive periportal access, CU may be a reasonable approach with no evident long‐term disadvantage. These findings support personalized surgical decision‐making rather than adherence to a one‐size‐fits‐all capsular protocol.

### Strengths and limitations

Strengths of this multilevel meta‐analysis include its rigorous methodological design, adherence to PRISMA guidelines, and use of a multilevel statistical framework suited for analyzing hierarchical outcome data. The inclusion of only mid‐ to long‐term studies addresses a key gap in the literature. However, limitations must be acknowledged. (1) The number of eligible studies was limited (*n* = 7), and all were conducted in the United States, potentially limiting generalizability. (2) Additionally, heterogeneity in surgical technique and rehabilitation protocols, as well as incomplete reporting of radiographic parameters (e.g., LCEA, alpha angle), may have introduced confounding. Nevertheless, this limitation should be acknowledged when interpreting between‐group differences, as the evidence base remains heterogeneous. (3) Three of the primary studies [31, 50, 53] contributed data to only one capsular technique, and none of them randomized capsular management. Within our multilevel meta‐analytic framework, such single‐arm contributions are statistically permissible and improve the precision of technique‐specific estimates without affecting the contrast structure between CP, CR and CU. (4) Furthermore, variation in other components of HAS (e.g., labral treatment, bony work, rehabilitation) across studies may have influenced outcomes beyond the capsular technique itself. (5) The available studies reported failure‐related outcomes inconsistently, which limited our ability to analyze hip survival and event‐free survival; therefore, long‐term success cannot be fully assessed based on PROMs alone. (6) Finally, publication bias cannot be entirely excluded, despite symmetric funnel plots for most outcomes.

### Future research

Further high‐quality, long‐term prospective studies—particularly randomized trials including both CP and CU arms—are needed to confirm these findings across diverse populations. The role of capsular management in subgroups such as borderline dysplasia, athletes or hypermobile individuals remains unclear and warrants targeted analysis. To comprehensively evaluate biomechanical restoration, postoperative follow‐up should include high‐resolution magnetic resonance imaging (MRI) to assess capsular integrity, focusing on capsule continuity and thickness. Additionally, the impact of capsular strategies on joint preservation and progression to THA beyond 10 years should be a focus of future work.

## CONCLUSION

Mid‐ to long‐term functional outcomes did not differ significantly between CP, CR and CU in this multilevel meta‐analysis. Failure‐related events (complications, reoperations, THA conversions) varied descriptively across techniques, but inconsistent reporting prevented firm conclusions regarding long‐term hip survivorship.

## AUTHOR CONTRIBUTIONS

Nikolai Ramadanov and Maximilian Voss performed the literature search, the data extraction and the risk of bias assessment. Robert Hable and Nikolai Ramadanov conducted the statistical calculations. Nikolai Ramadanov and Robert Hable created all figures and tables. Nikolai Ramadanov wrote the manuscript. Ingo J. Banke, Robert Prill and Roland Becker supervised the work.

## CONFLICT OF INTEREST STATEMENT

The authors declare no conflicts of interest.

## ETHICS STATEMENT

Ethical approval was not required for this study, as it is a systematic review and multilevel meta‐analysis of previously published data.

## Supporting information

Supplementary_Table_1.

## Data Availability

Data are available from the corresponding author upon reasonable request.
